# Differential Processing of a *Bacillus subtilis* GH5 Endoglucanase During Yeast Surface Display in *Saccharomyces cerevisiae*

**DOI:** 10.3390/microorganisms14051061

**Published:** 2026-05-08

**Authors:** Joel Ríos-Alvarado, Perla Guadalupe Vázquez-Ortega, Norma Urtiz-Estrada, Javier López-Miranda, Jesús Bernardo Páez-Lerma, María Adriana Martínez-Prado, Marcelo Barraza-Salas, David Enrique Zazueta-Álvarez, Damián Reyes-Jáquez, Alma Karina Tamez-Castrellón, Juan Antonio Rojas-Contreras

**Affiliations:** 1 Departamento de Ingenierías Química y Bioquímica, Tecnológico Nacional de México/Instituto Tecnológico de Durango, Blvd. Felipe Pescador 1830 Ote., Durango 34080, Mexico; 7790027@itdurango.edu.mx (J.R.-A.); jlopez@itdurango.edu.mx (J.L.-M.); jpaez@itdurango.edu.mx (J.B.P.-L.); adriana.martinezprado@itdurango.edu.mx (M.A.M.-P.); damian.reyes@itdurango.edu.mx (D.R.-J.); soulk_taca@hotmail.com (A.K.T.-C.); 2 Facultad de Ciencias Químicas, Universidad Juárez del Estado de Durango, Av. Veterinaria s/n, Circuito Universitario, Col. Valle del Sur, Durango 34120, Mexico; norma.urtiz@ujed.mx (N.U.-E.); mbsalas@ujed.mx (M.B.-S.); 3 Departamento de Ingeniería en Tecnología Ambiental, Universidad Politécnica de Durango, Carretera Durango-México km. 9.5, Durango 34300, Mexico; david.zazueta@unipolidgo.edu.mx

**Keywords:** yeast surface display, GH5 endoglucanase, heterologous expression, cellulase, whole-cell biocatalyst

## Abstract

Yeast surface display is a powerful strategy for enzyme immobilization and whole-cell biocatalysis; however, the intracellular processing of heterologous enzymes during secretion and anchoring remains poorly understood. In this study, a GH5 endoglucanase gene (*egl*S, 1.4 kb) from *Bacillus subtilis*, originally isolated from a paper mill effluent, was cloned into the pYD1 vector and expressed in *Saccharomyces cerevisiae* EBY100 using the Aga1–Aga2 surface display system. The recombinant strain produced clear degradation halos on carboxymethyl cellulose (CMC) plates, confirming cellulolytic activity at the whole-cell level. Zymographic analysis revealed multiple active enzyme forms depending on the cellular fraction analyzed. Intracellular extracts displayed active bands ranging from 70 to 57 kDa, consistent with immature or partially processed Aga2 fusion proteins, whereas cell wall-associated fractions showed active bands between 55 and 35 kDa, suggesting proteolytic processing during secretion and surface anchoring. The apparent specific activity of the cytoplasmic fraction was 5.33 ± 0.31 U mg^−1^, while the cell wall-associated fraction exhibited a higher apparent specific activity (58.4 ± 10.1 U mg^−1^). Although these values were obtained from non-purified fractions and therefore do not represent intrinsic enzymatic constants, they indicate a relative enrichment of catalytically active enzyme in the cell wall-associated fraction, consistent with functional surface display. The presence of multiple active enzyme forms and the enhanced catalytic efficiency observed in the cell wall-associated fraction suggest that the engineered yeast strain may serve as a promising whole-cell biocatalyst, with potential applications in consolidated bioprocessing (CBP) strategies for lignocellulosic biomass conversion.

## 1. Introduction

Cellulases are utilized in various industries, including food production; beer and wine manufacturing; textile and laundry sectors; animal feed; pulp and paper treatment; agriculture; waste management; pharmaceuticals; and biofuel production [1]. Biofuels are becoming increasingly important in developing countries, and research efforts have focused on technologies for producing second-generation (2G) bioethanol from lignocellulosic or non-food biomass [2]. These feedstocks are inexpensive and mainly comprise cellulose (30–60%), hemicellulose (20–40%), and lignin (20–30%) [3]. Renewable sources for bioethanol production include agricultural waste, lignocellulosic biomass, energy crops, and forest residues [4]. Bioethanol production involves pretreating biomass using physical, chemical, or combined methods, followed by enzymatic hydrolysis with cellulases to increase fermentable sugars, and concluding with fermentation by ethanologenic yeast and purification through distillation [5,6]. Three enzymes are required to transform cellulose into fermentable sugars: endoglucanase, which hydrolyzes internal bonds in cellulose chains; cellobiohydrolase, which degrades and produces cellobiose and cyclodextrin units from chain ends; and β-glucosidase, which converts cellodextrin and cellobiose into glucose [7,8]. Cellulases play a crucial role in the production of cellulosic ethanol at a lower cost. Although the costs of cellulase production have decreased, it remains an economic challenge [9,10]. A viable approach to lower ethanol production costs involves either engineering non-cellulolytic ethanol producers, like *Saccharomyces cerevisiae*, to produce cellulases or modifying cellulase producers to generate ethanol. This method, known as consolidated bioprocessing (CBP), integrates all biological processes required to convert lignocellulosic biomass into ethanol [11].

Efficient heterologous production of cellulolytic enzymes in *S. cerevisiae* requires careful consideration of codon usage bias, as well as co- and post-translational events that influence protein folding, stability, and activity [12]. In particular, glycosylation has been reported to negatively affect the activity of cellulases such as Cel6A and Cel7A from *Trichoderma reesei* when expressed in recombinant hosts [13].

The heterologous production and functional display of cellulases in yeast using yeast surface display systems have been previously demonstrated, highlighting their potential for direct cellulose hydrolysis and consolidated bioprocessing applications [12,14,15]. However, these studies have mainly focused on overall enzymatic performance, with limited attention to the intracellular processing, post-translational modifications, and differential localization of the recombinant enzymes.

In particular, the behavior of GH5 endoglucanases from *Bacillus subtilis* in yeast expression systems remains poorly understood, especially regarding their processing, retention on the cell surface, and potential release into the extracellular medium.

To address this gap, the *egl*S gene, encoding a β-1,4-endoglucanase derived from a *Bacillus subtilis* strain isolated from a paper mill effluent, was expressed in *S. cerevisiae* using a yeast surface display system. This enzyme was previously produced in *Escherichia coli* as a truncated, monomodular, and highly processive endoglucanase lacking a carbohydrate-binding module (CBM) as reported in a previous study from our group [16], suggesting that truncation may be an intrinsic feature of this enzyme rather than a host-specific artifact.

In this study, we aim to evaluate the enzymatic activity and overall behavior of this enzyme when heterologously expressed in *S. cerevisiae*, with particular emphasis on its processing and distribution. While most previous studies have focused on maximizing enzyme production and overall activity, comparatively little attention has been paid to the intracellular processing events that ultimately determine the functional outcome of heterologous expression systems.

In addition to assessing the potential of the engineered *S. cerevisiae* strain as a whole-cell biocatalyst for second-generation (2G) bioethanol production through consolidated bioprocessing (CBP), this work provides new insights into how bacterial endoglucanases are processed and function within yeast surface display systems.

## 2. Materials and Methods

### 2.1. Plasmids, Strains and Media

The microbial strains and plasmids used in this study are listed in Table 1. *Escherichia coli* strain JARC03 was cultured in low-salt LB broth (Lennox formulation), and ampicillin was added at a final concentration of 100 µg mL^−1^ for selection. *Saccharomyces cerevisiae* EBY100 (trp1, leu2Δ,) was grown in yeast nitrogen base (YNB) medium supplemented with 0.01% (*w*/*v*) leucine and 0.01% (*w*/*v*) tryptophan. For plasmid maintenance and verification of EBY100 strain transformation, selective YNB medium lacking tryptophan was used.

For plate-based expression and cellulolytic activity screening, yeast cells were grown at 27 °C on YNB agar supplemented with 0.5% (*w*/*v*) casamino acids (CAAs), 0.01% (*w*/*v*) leucine, 2% (*w*/*v*) galactose as an inducer, and 1% (*w*/*v*) carboxymethylcellulose (CMC) as substrate.

### 2.2. Reagents

LB broth, yeast nitrogen base (YNB), casamino acids (CAAs), sodium carboxymethyl cellulose (medium viscosity, CMC), galactose, tryptophan, Congo Red, and ampicillin were purchased from Sigma-Aldrich (St. Louis, MO, USA). The pGEM-T Easy Vector, DNA restriction enzymes, T4 DNA ligase, and Taq DNA polymerase were obtained from Promega (Madison, WI, USA). Tris base, acrylamide, bis-acrylamide, glycine, molecular weight standards, and electrophoresis reagents were purchased from Bio-Rad Laboratories (Hercules, CA, USA).

### 2.3. Cloning of B. subtilis eglS Gene and Strains Transformation

The *egl*S ORF from *B. subtilis* was amplified as a 1.4 kb fragment from pITD03 (Table 1) using the primer pair *egl*S-D (5′-GGATCCACGCCAGTAGCCAAGAATGGC-3′) and *egl*S-R (5′-GCGGCCGCATGGTTCTGTTCCCCAAATCAGTTTTCC-3′). The amplified fragment was cloned into the pGEM-T Easy plasmid and then subcloned into the *BamH*I-*Not*I restriction sites of the pYD1 plasmid using T4 DNA ligase according to the supplier’s protocol (pYD1 was a gift from Dane Wittrup; Addgene plasmid # 73447; http://n2t.net/addgene:73447; RRID: Addgene_73447). Transformation of *Escherichia coli* DH5α was carried out using competent cells using the transformation method described previously [19]. Recombinant strains were selected on LB medium containing ampicillin. After transformation, plasmid construction was confirmed using enzyme restriction assay and 1% agarose gel electrophoresis stained with ethidium bromide (EtBr). The constructed plasmid, named pITD06 (Table 1), was utilized for the transformation of *S. cerevisiae* strain EBY100 using the small-scale lithium acetate (LiAc) yeast chemical transformation protocol as described previously [20]. To corroborate the transformation of the *S. cerevisiae* EBY100, the parental and transformed strains were streaked on plates containing YNB medium without tryptophan and incubated at 28 °C for 48 h. The presence of pITD06 in the *S. cerevisiae* EBY100 was confirmed by colony PCR of the transformed yeast using the primer pair 5′-CAGCAAATGGGGGTCGGGGGATCT-3′ and reverse primer 5′-CGAGACCGAGGAGGAGAGAGAGGGTTA-3′), flanking the multiple cloning site (MCS) of the pYD1 plasmid.

### 2.4. Evaluation of Cellulase Activity in Recombinant S. cerevisiae

To evaluate cellulolytic activity, *S. cerevisiae* EBY100 transformed with the pITD06 plasmid carrying the *egl*S gene (designated *S. cerevisiae* JARC06) and the control strain harboring the empty pYD1 vector were grown on YNB agar plates supplemented with 1% (*w*/*v*) CMC. Plates were incubated at 27 °C and analyzed after 24, 48, and 72 h of growth.

Cellulolytic activity was visualized by staining the plates with 0.25% (*w*/*v*) Congo Red solution for 30 min, followed by washing with 1 M NaCl solution for 10 min to reveal zones of CMC hydrolysis as clear halos against a stained background.

### 2.5. Induction of Recombinant β-1,4-Endoglucanase Expression in S. cerevisiae

Yeast surface display and induction were performed following the protocol described for the pYD1 yeast display system [21], including galactose induction at reduced temperature to improve protein folding. The *S. cerevisiae* JARC06 strain was cultured overnight at 30 °C in 10 mL of YNB medium supplemented with 0.5% (*w*/*v*) casamino acids (CAAs), 0.01% (*w*/*v*) leucine, and 2% (*w*/*v*) glucose under agitation at 180 rpm. Cells were then centrifuged at 3000× *g* for 5 min at room temperature, and the supernatant was discarded. For induction, the cell pellet was resuspended in 50 mL of YNB medium containing 2% (*w*/*v*) galactose to an OD_600_ of 1.0. As a control, the *S. cerevisiae* EBY100 strain harboring the empty pYD1 vector was cultured under the same conditions. Cultures were incubated at 22 °C for 36 h. Subsequently, cultures were centrifuged at 3000× *g* for 10 min at 4 °C to separate the cell pellet and culture supernatant. Both fractions were collected for further analysis.

### 2.6. Enzyme Extraction from Different Cellular Fractions

For the analysis of cell wall-anchored proteins, the cell pellet obtained after centrifugation at 2500× *g* for 15 min at 4 °C, was washed three times with 5 mL of 1× PBS (137 mM NaCl, 2.7 mM KCl, 10 mM Na_2_HPO_4_, 1.8 mM KH_2_PO_4_, pH 7.4). The washed pellet was then resuspended in 5 mL of 1× PBS containing Dithiothreitol (DTT) 100 µM and incubated under agitation at 300 rpm for 2 h to release proteins anchored to the cell wall through disulfide bonds. Cells were subsequently separated from the supernatant by centrifugation, and the recovered supernatant was stored for further analysis of the cell wall-associated enzyme fraction.

To analyze the proteins secreted into the culture medium, the supernatant obtained in the previous step was collected and concentrated 100-fold (150 µL from an initial volume of 15 mL) by centrifugation at 2500× *g* for 60 min at 4 °C using a Millipore Centricon ultrafiltration device (Darmstadt, Germany) equipped with a 10 kDa molecular weight cutoff membrane.

The remaining cell pellet was washed three times with 5 mL of 1× PBS, centrifuged as described above, and resuspended in 5 mL of cell disruption buffer (50 mM sodium phosphate buffer, pH 7.4, 1 mM EDTA, and 5% glycerol). An equal volume of acid-washed glass beads (0.5 mm) was then added. Cells were disrupted by vortexing for 30 s, followed by incubation on ice for 30 s; this cycle was repeated eight times. The lysate was centrifuged at 2500× *g* for 30 min at 4 °C, and the clear supernatant containing intracellular proteins was transferred to fresh microcentrifuge tubes.

### 2.7. Protein Determination and Zymogram Analysis of Endoglucanase Activity

Protein concentrations in the different enzyme extracts were determined using the Bradford method with the Coomassie Plus™ Protein Assay Kit (Thermo Scientific, Carlsbad, CA, USA) [22], using bovine serum albumin (BSA) as the standard.

For the initial zymogram assays, enzyme extracts and control samples (protein extracts obtained from the parental EBY100 strain) were prepared by mixing 10 µL of each extract with 5 µL of 2× SDS–PAGE sample loading buffer without β-mercaptoethanol and without prior heating. Under these conditions, zymogram analysis revealed diffuse comet-like activity patterns rather than well-defined bands. To improve band resolution, samples were subsequently prepared using 2× SDS–PAGE loading buffer containing β-mercaptoethanol and heated at 95 °C for 5 min prior to electrophoresis.

An 8% (*w*/*v*) polyacrylamide gel copolymerized with 0.5% (*w*/*v*) carboxymethylcellulose (CMC) was used for endoglucanase zymogram analysis. Electrophoresis was conducted in Tris–glycine running buffer (25 mM Tris, 192 mM glycine, 0.1% (*w*/*v*) SDS, pH ~ 8.3) at 100 V for approximately 120 min. After electrophoresis, gels were either washed twice with 2.5% (*v*/*v*) Triton X-100 in distilled water at 20 °C for 30 min to remove SDS and allow partial renaturation or directly incubated in renaturation buffer. In both cases, enzymatic activity was detected, indicating that the enzyme retains sufficient activity under the assay conditions.

The gel was then incubated in 50 mM sodium acetate buffer at 50 °C for 12 h to allow enzymatic hydrolysis of the substrate. Subsequently, the gel was stained with 0.1% (*w*/*v*) Congo Red solution for 15 min and destained twice with 1 M NaCl for 15 min. Hydrolytic activity appeared as clear bands against a dark red background. In parallel, an SDS–PAGE gel with the same composition as that used for the zymogram was run and stained with Coomassie Brilliant Blue G-250 to visualize the protein banding pattern and compare the molecular sizes of the bands associated with the enzymatic activity detected in the zymogram.

A prestained protein molecular weight marker ranging from 10 to 245 kDa (Bio-Rad) was used as a size reference.

### 2.8. Determination of Endoglucanase Activity

Endoglucanase activity was determined by measuring the amount of reducing sugars released from carboxymethylcellulose (CMC) using the dinitrosalicylic acid (DNS) method described by Miller [23].

The reaction mixture consisted of 225 µL of 50 mM sodium acetate buffer (pH 5.0), 225 µL of 1% (*w*/*v*) CMC, and 50 µL of enzyme extract, resulting in a final reaction volume of 500 µL. The mixture was incubated at 50 °C for 20 min.

The enzymatic reaction was stopped by adding 500 µL of DNS reagent, followed by incubation in boiling water for 5 min to allow color development. The reaction tubes were then cooled in an ice bath to stop further color formation.

The absorbance was measured at 540 nm using a UV–Vis DR 6000 HACH spectrophotometer (Hach Company, Loveland, CO, USA). The concentration of reducing sugars released was calculated from a glucose standard curve prepared under the same conditions.

One unit (U) of enzyme activity was defined as the amount of enzyme required to release 1 µmol of glucose equivalents per minute under the assay conditions.

All enzymatic assays were performed in triplicate, and the results are reported as mean values.

## 3. Results

### 3.1. Cloning of the β-1,4-Endoglucanase Gene in S. cerevisiae EBY100

**Figure 1:**
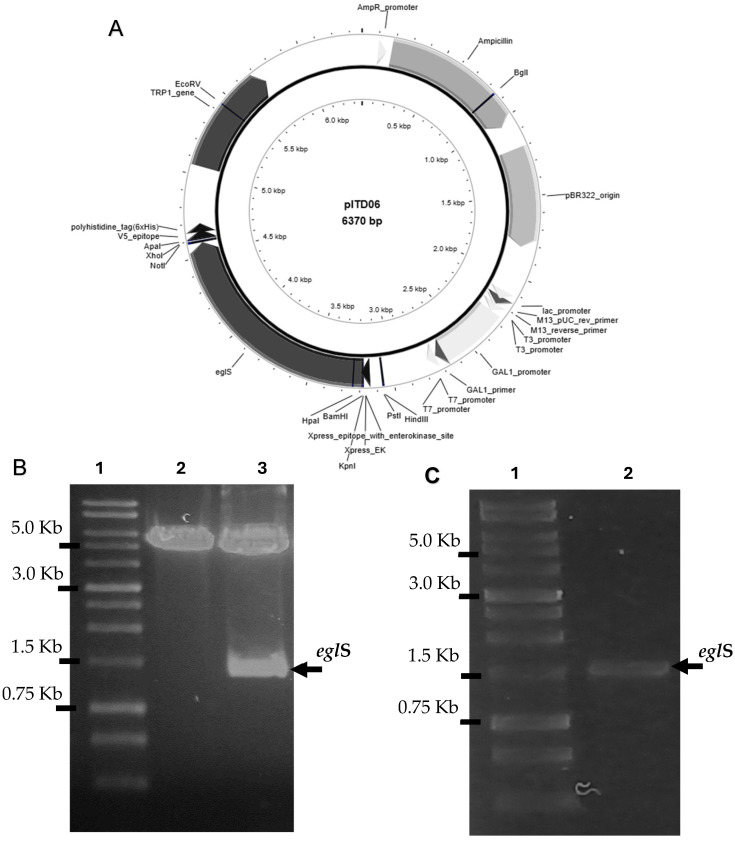
(**A**) pITD06 vector map. Key features are indicated with arrows clockwise: Amp^r^ (ampicillin resistance for *E. coli* selection), pBR322 origin of replication, GAL1 promoter (galactose-inducible expression), *egl*S gene (*Bam*HI/*Not*I), and TRP1 (selection in *Saccharomyces cerevisiae* EBY100 via tryptophan auxotrophy complementation). (**B**) Restriction analysis of pITD06 using *Bam*HI and *Not*I enzymes; lane 1, 1 kb DNA ladder; lane 2, empty pYD1 plasmid; lane 3, pYD1 carrying the 1.4 kb *egl*S insert (pITD06). (**C**) PCR confirmation of the *egl*S insertion into pITD06; lane 1, 1 kb DNA ladder; lane 2, 1.4 kb amplicon corresponding to *egl*S. The plasmid map was created using PlasMapper 3.0 [24].

The *egl*S gene sequence lacking its signal peptide was amplified by PCR using the plasmid pITD03, which was previously reported by Ríos et al. (2024) [16], as template. This plasmid harbors the *egl*S gene from *Bacillus subtilis*, which encodes a β-1,4-endoglucanase (EglS) with high enzymatic activity. The resulting PCR product (approximately 1.4 kb in length) was ligated into the *Bam*HI and *Not*I restriction sites of the pYD1 expression vector, and the resulting recombinant plasmid was designated pITD06 (Figure 1A). Restriction analysis using the corresponding enzymes confirmed the successful insertion of the *egl*S gene (Figure 1B). Additionally, PCR amplification using primers flanking the multiple cloning site (MCS) of pITD06 further verified the correct insertion of the gene, yielding the expected amplification product (Figure 1C). The pITD06 plasmid enables the galactose-regulated expression, secretion, and surface display of EglS on the extracellular surface of *S. cerevisiae.* Secretion and anchoring of cellulase to the yeast cell wall are mediated by its fusion to the Aga2p subunit of the α-agglutinin mating receptor. In the *S. cerevisiae* EBY100 strain, the complementary Aga1p subunit is constitutively integrated into the genome and expressed under the control of a galactose-inducible (GAL) promoter. Upon galactose induction, Aga1p is produced and forms disulfide bonds with the Aga2p–cellulase fusion protein, thereby enabling stable localization and display of the EglS endoglucanase on the yeast cell surface.

### 3.2. Detection of EglS Endoglucanase Activity on CMC Agar Plates

**Figure 2:**
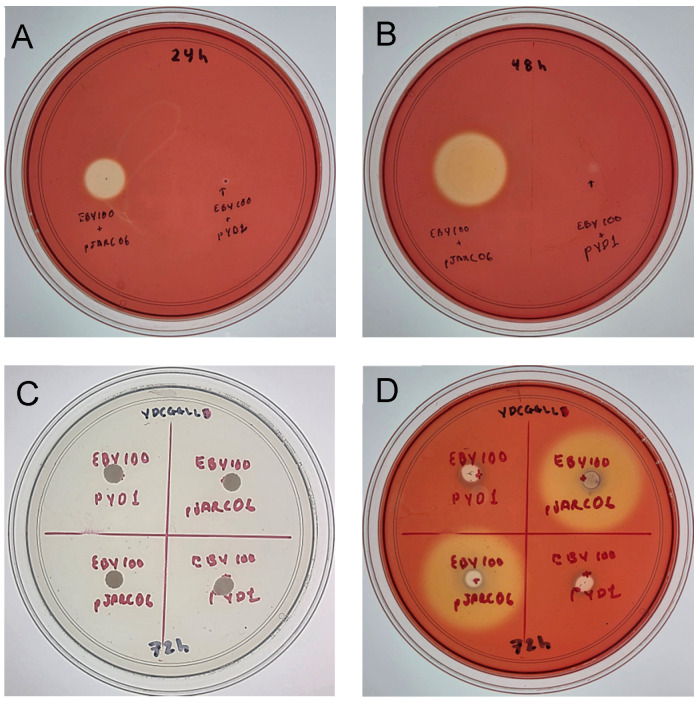
Cellulase activity assay of *Saccharomyces cerevisiae* strains on YNB agar plates supplemented with 1% (*w*/*v*) carboxymethyl cellulose (CMC) incubated at 27 °C. (**A**,**B**) Growth of the control strain (*S. cerevisiae* EBY100 harboring the empty pYD1 vector) and the recombinant strain expressing *egl*S (JARC06) after 24 h and 48 h of incubation, respectively. (**C**) Plate after 72 h of incubation prior to staining. (**D**) The same plate after Congo Red staining to visualize cellulose hydrolysis. Comparable growth was observed between control and recombinant strains at all time points.

Cellulase activity was initially evaluated on YNB agar plates supplemented with 1% (*w*/*v*) carboxymethyl cellulose (CMC) using Congo Red staining. To provide a comparative assessment and address potential differences in growth, both the control strain (*S. cerevisiae* EBY100 harboring the empty pYD1 vector) and the recombinant strain expressing *egl*S (JARC06) were analyzed at 24 h and 48 h of incubation (Figure 2A,B). At both time points, the two strains exhibited comparable growth; however, a hydrolysis halo was observed only in the recombinant strain JARC06, whereas no halo was detected in the control strain carrying the empty vector.

After 72 h of incubation, plates were examined prior to staining (Figure 2C), confirming similar growth between strains. Following Congo Red staining (Figure 2D), a clear hydrolysis halo was again observed only in the recombinant strain expressing *egl*S, whereas no halo was detected in the control strain. The presence of these halos suggests that part of the recombinant cellulase may reach the culture medium, allowing the enzyme to interact with the insoluble substrate surrounding the colonies. Although the Aga1–Aga2 system is designed to anchor recombinant proteins to the yeast cell wall, the formation of degradation halos may indicate that a fraction of the enzyme is released into the extracellular environment or remains sufficiently exposed at the cell surface to hydrolyze the substrate.

### 3.3. Zymographic Analysis of Recombinant Endoglucanase Localization in Different Cellular Fractions

To investigate the localization and processing of the recombinant EglS endoglucanase in *S. cerevisiae*, zymogram analysis was performed on the enzyme extracts obtained from the culture supernatant, the cell wall-associated fraction released after DTT treatment, and the intracellular fraction obtained after cell disruption. In parallel, SDS–PAGE gels with the same polyacrylamide composition were run and stained with Coomassie Brilliant Blue G-250 to visualize the protein-banding pattern and compare the apparent molecular weights of the bands associated with enzymatic activity.

**Figure 3:**
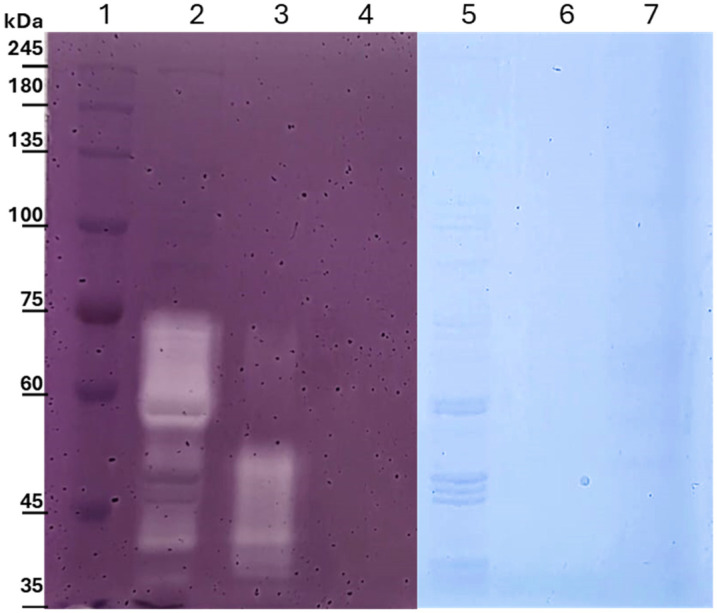
Zymographic analysis of recombinant endoglucanase activity in different cellular fractions of *Saccharomyces cerevisiae* JARC06. Lane 1 shows the prestained protein molecular weight marker. Lane 2 shows the zymogram of the intracellular (cytoplasmic) enzyme extract. Lane 3 shows the zymogram of the cell wall-associated enzyme fraction released after DTT treatment. Lane 4 shows the zymogram of the enzyme fraction secreted into the culture medium. Lanes 5–7 show SDS–PAGE gel runs parallel with the zymogram and stained with Coomassie Brilliant Blue G-250 to visualize the protein-banding pattern. Lane 5 shows the cytoplasmic protein fraction, lane 6 shows the cell wall-associated proteins, and lane 7 shows the proteins present in the culture supernatant.

As shown in Figure 3, zymogram analysis revealed the presence of multiple cellulolytic activity bands depending on the cellular fraction analyzed. In the intracellular fraction, several activity bands were detected within a molecular weight range of approximately 73 to 57 kDa. These bands likely correspond to intermediate forms of the recombinant fusion protein during its processing through the secretory pathway. Considering the predicted molecular weight of the recombinant construct and the presence of the Aga2 fusion partner, these higher molecular weight bands may represent immature or partially processed forms of the enzyme that still contain signal peptide remnants, Aga2 sequences, or other domains involved in the anchoring process.

In contrast, the cell wall-associated fraction displayed activity bands with apparent molecular weights ranging from approximately 55 to 37 kDa. The band near 55 kDa is consistent with the expected size of the EglS catalytic domain containing its carbohydrate-binding module (CBM) fused to Aga2 after removal of the signal peptide, suggesting successful anchoring of the recombinant enzyme at the yeast cell surface. The lower molecular weight bands observed between 37 and 35 kDa likely correspond to truncated forms of the enzyme generated by proteolytic processing during secretion or after cell wall anchoring. Similar processing patterns have been reported for heterologously expressed cellulases in *S. cerevisiae*, where partial proteolysis or differential glycosylation can generate multiple active isoforms of the same enzyme [25]. Interestingly, in a previous study from our group, the same EglS enzyme produced in a secretable form in *Escherichia coli* was shown to undergo a self-truncation process characteristic of certain GH5 cellulases, resulting in a monomodular enzyme lacking the CBM domain [16]. In that context, the activity band observed around 37 kDa in the present study is consistent with the expected size of this truncated monomodular form, suggesting that a similar processing event may occur during heterologous expression in *S. cerevisiae*.

Interestingly, little to no defined cellulolytic activity bands were detected in the concentrated culture supernatant despite the clear degradation halos observed in plate assays. This observation suggests that most enzymatic activity remains associated with the yeast cell surface or intracellular compartments, although a small fraction of the enzyme may diffuse into the surrounding medium during growth.

### 3.4. Zymographic Analysis of Endoglucanase Activity in Cytoplasmic Extracts

**Figure 4:**
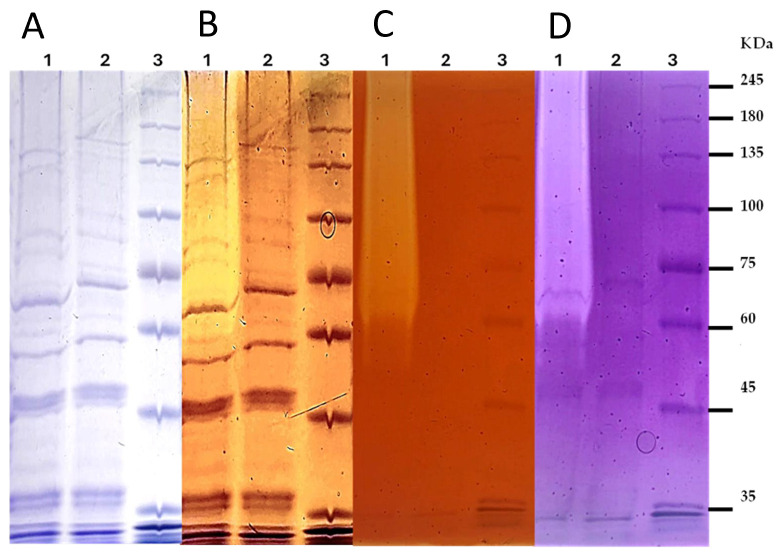
*Zymographic analysis of endoglucanase activity in cytoplasmic extracts of Saccharomyces cerevisiae.* SDS–PAGE gels copolymerized with 0.5% (*w*/*v*) carboxymethyl cellulose (CMC) were used to evaluate enzymatic activity. Cytoplasmic extracts from the recombinant strain expressing *egl*S and the control strain (*S. cerevisiae* EBY100 harboring the empty pYD1 vector) were analyzed under identical conditions. Lanes: (1) recombinant strain, (2) control strain, (3) molecular weight marker. (**A**,**B**) Same gel: (**A**) Coomassie Brilliant Blue staining and (**B**) Congo Red staining showing hydrolytic activity as clear zones. (**C**,**D**) Independent replicate: (**C**) Congo Red staining and (**D**) subsequent Coomassie staining. Hydrolytic activity is detected exclusively in the recombinant strain.

Zymographic analysis was performed to evaluate the presence and distribution of endoglucanase activity in cytoplasmic extracts of *S. cerevisiae*. As shown in Figure 4, clear hydrolytic bands were observed exclusively in the recombinant strain expressing *egl*S, while no activity was detected in the control strain harboring the empty pYD1 vector despite comparable protein loading.

To assess both protein profile and enzymatic activity, gels copolymerized with CMC were subjected to sequential staining. In one set of experiments (Figure 4A,B), Coomassie Brilliant Blue staining allowed visualization of protein-band patterns, followed by Congo Red staining of the same gel to detect zones of CMC hydrolysis. In this case, hydrolytic activity was detected only in the recombinant strain.

In a second independent replicate (Figure 4C,D), the gel was first stained with Congo Red to enhance visualization of enzymatic activity, revealing more clearly defined hydrolytic zones. Subsequent Coomassie staining of the same gel enabled partial visualization of protein bands, although with reduced clarity compared to direct Coomassie staining.

Although differences in band visibility were observed depending on the staining sequence, consistent detection of activity exclusively in the recombinant strain across independent gels confirms that the observed cellulolytic activity is specifically associated with *egl*S expression. These results also suggest the presence of active enzyme forms in the cytoplasmic fraction, including lower apparent molecular weight species, likely corresponding to processed forms of the recombinant protein.

The absence of detectable activity in the control strain further supports that the observed hydrolytic bands are not due to endogenous yeast enzymes.

### 3.5. Comparative Enzymatic Activity of Recombinant EglS in Different Cellular Fractions

Enzymatic activity assays revealed that the cytoplasmic fraction exhibited the highest volumetric activity, reaching 10.52 ± 0.13 U mL^−1^ of extract, whereas the cell wall-associated fraction showed a lower volumetric activity of 3.79 ± 0.04 U mL^−1^ of extract. These values were calculated from the activity measured using 50 µL of each extract and extrapolated to 1 mL of extract; therefore, they represent activity concentration within each fraction. Considering the final recovered volume of each fraction (5 mL), the corresponding total activities were 52.6 ± 0.7 U for the cytoplasmic fraction and 18.95 ± 0.20 U for the cell wall-associated fraction. When normalized to protein content, the wall-associated fraction displayed a markedly higher apparent specific activity (58.4 ± 10.1 U mg^−1^) than the cytoplasmic extract (5.33 ± 0.31 U mg^−1^), indicating an approximately 10.9-fold enrichment in catalytically active enzyme. Whole recombinant cells also retained detectable hydrolytic activity, reaching 0.0795 U per assay, supporting the functionality of the displayed enzyme in the intact cell context. A summary of the enzymatic activities and calculated parameters for each fraction is presented in Table 2.

## 4. Discussion

Biochemical characterization of the different cellular fractions revealed clear differences in enzymatic performance depending on the cellular localization of the recombinant endoglucanase expressed in *Saccharomyces cerevisiae*. As expected, the cytoplasmic extract displayed the highest volumetric activity (10.52 ± 0.13 U mL^−1^), which can be attributed to the relatively high total protein concentration detected in this fraction (1.97 ± 0.110 mg mL^−1^). However, because this fraction also contains a substantial number of endogenous yeast proteins released during cell disruption, its specific activity remained comparatively low (5.33 ± 0.31 U mg^−1^). This observation indicates that the recombinant enzyme represents only a fraction of the soluble protein pool recovered after cell lysis, a common limitation in heterologous protein expression systems [26,27].

In contrast, the cell wall-associated fraction displayed markedly higher catalytic efficiency when normalized to the protein content. Although its volumetric activity was lower (3.79 ± 0.04 U mL^−1^), the apparent specific activity reached 58.4 ± 10.1 U mg^−1^, corresponding to an approximately 11-fold enrichment relative to the cytoplasmic fraction. This enrichment strongly supports the effective localization of the recombinant enzyme at the cell surface via the Aga1–Aga2 anchoring system. Yeast surface display platforms are well-established tools for protein engineering and biocatalysis, and have been widely used to display enzymes involved in biomass degradation [28,29]. In such systems, anchoring enzymes to the yeast cell wall reduces background contamination from intracellular proteins while simultaneously enabling direct contact between the catalytic domain and polymeric substrates.

A similar observation has been reported by Mormeneo et al. (2012) [25], who expressed the endoglucanase CelA from *Paenibacillus barcinonensis* in *S. cerevisiae* using Pir4 fusion constructs designed for either secretion or cell wall retention. Although the cell wall-targeted construct was expected to retain the enzyme at the yeast surface, cellulase activity was also detected in the culture medium and in plate assays, suggesting partial leakage or release of the cell wall-associated fusion protein into the growth medium. This behavior is consistent with the results observed in the present study and indicates that, even in surface display systems, complete retention of the enzyme at the cell wall is not always achieved.

Beyond this previously reported behavior, the present study provides additional insight into the functional consequences of enzyme localization. In particular, our results indicate that multiple processed forms of the enzyme remain catalytically active, suggesting that proteolytic processing or self-truncation does not necessarily compromise enzymatic function. However, the precise origin and mechanism of these processed forms cannot be conclusively determined based on the present data.

The catalytic competence of the enzyme after cell wall anchoring is consistent with the biochemical properties previously reported for this enzyme, including high tolerance to variations in temperature and pH [16]. During zymographic analysis, it was necessary to heat the samples for 5 min in the presence of reducing agents prior to electrophoresis in order to suppress enzymatic activity during gel migration. Without this treatment, the enzyme remained catalytically active during electrophoresis, producing a comet-like pattern caused by continuous substrate degradation along the migration path. The requirement of this step to obtain discrete activity bands highlights the remarkable stability of the enzyme and suggests the presence of a structurally robust catalytic domain.

Such behavior is consistent with the structural characteristics of glycosyl hydrolase family 5 (GH5) enzymes, which typically adopt compact (β/α)_8_ barrel folds associated with high structural stability [30,31]. Indeed, previous studies have demonstrated that several endoglucanases from bacterial and fungal sources can retain or recover activity after exposure to denaturing conditions. For example, structural and functional analyses of cellulases from *Trichoderma reesei* have shown that the catalytic core can maintain activity following thermal perturbation when its overall fold remains intact [32]. Similarly, studies on bacterial GH5 enzymes have highlighted the relationship between structural rigidity and catalytic resilience [30]. These observations support the interpretation that the enzymatic activity detected after electrophoretic treatment is not an experimental artifact but instead reflects the intrinsic stability of the catalytic domain.

Another feature that may explain the behavior observed in the wall-associated fraction is the self-truncation phenomenon previously described for this enzyme [16]. Previous studies have reported that certain endoglucanases undergo spontaneous processing, generating shorter catalytically active forms lacking accessory domains such as carbohydrate-binding modules (CBMs) [33]. In the context of yeast surface display, it is plausible that partial proteolytic processing occurs after secretion or during cell wall anchoring, generating multiple truncated but catalytically active fragments that remain associated with the yeast surface.

Although proteolytic processing is often considered undesirable during heterologous protein expression, in this case it may represent a potential functional advantage. Several studies have demonstrated that monomodular endoglucanases lacking CBMs can display improved catalytic efficiency toward soluble cellulose derivatives, likely due to reduced steric constraints and improved accessibility of the catalytic domain to the substrate [34,35].

An important technical consideration in the interpretation of zymographic and SDS–PAGE analyses is the inherent limitation in protein detection sensitivity. In the present study, the protein content of the cell wall-associated and extracellular fractions was relatively low, making it difficult to achieve equivalent protein loading across samples. Visualization of bands by Coomassie staining typically requires microgram-level quantities of protein per lane, which would necessitate extensive concentration of large volumes of culture supernatant or cell wall extracts. As a result, discrepancies in band intensity between samples should not be interpreted as strictly quantitative differences in enzyme abundance. Instead, zymograms provide a qualitative or semi-quantitative assessment of enzymatic activity, allowing detection of active species even when total protein is below the threshold of conventional staining methods.

In addition to the activity detected in isolated fractions, intact recombinant yeast cells exhibited measurable cellulolytic activity, confirming that the enzyme remains accessible and catalytically functional when displayed on the yeast surface. Although this behavior could be interpreted as a limitation from an application perspective, this depends on the intended application. In consolidated bioprocessing (CBP) and related lignocellulose-based processes, the use of intact recombinant yeast cells as whole-cell biocatalysts offers significant advantages, such as eliminating enzyme purification, simplifying catalyst recovery, and enabling the coupling of hydrolysis and fermentation in a single system [36,37,38].

Such systems are particularly attractive for CBP approaches aimed at converting lignocellulosic biomass into fermentable sugars. In this context, it is important to note that significantly higher cellulase activities have been reported in heterologous expression systems specifically optimized for enzyme production and secretion, particularly in *Pichia pastoris*. In these systems, endoglucanase activities in the range of hundreds to thousands of U mL^−1^ have been achieved under optimized fermentation conditions and, in many cases, following purification [39,40,41]. However, these systems are designed to maximize enzyme yield and are not directly comparable to surface display platforms. In contrast, the present study focuses on enzyme functionality within a cell-associated system, where enzymatic activity is distributed across cellular compartments and measured in complex mixtures. Therefore, direct comparison of activity values is not straightforward, and the results should instead be interpreted in terms of enzyme localization, processing, and functional accessibility.

Accordingly, the primary objective of the present study was to evaluate the functional expression, processing, and distribution of the enzyme in a yeast surface display system rather than to maximize enzyme production. Future optimization of this system could involve the co-expression of complementary cellulolytic enzymes and the development of multi-enzyme display platforms for CBP applications.

In summary, the results presented here demonstrate that the recombinant endoglucanase retains catalytic activity across multiple cellular contexts and remains functional even after exposure to conditions typically associated with protein denaturation. The combination of structural robustness, tolerance to processing, and compatibility with yeast surface display highlights the potential of this enzyme as a promising component for the development of yeast-based lignocellulose bioconversion platforms.

## 5. Conclusions

Recombinant endoglucanase EglS expressed in *Saccharomyces cerevisiae* remained catalytically active in cytoplasmic, cell wall-associated, and whole-cell contexts. Surface display via the Aga1–Aga2 system enriched the enzyme at the yeast cell wall, resulting in a markedly higher specific activity than that of the cytoplasmic extract.

The enzyme even maintained activity after the thermal treatment required for zymographic analysis, supporting previous reports of its high stability to temperature and pH. In addition, the previously described self-truncation of EglS may generate multiple active catalytic fragments at the cell surface, which could contribute to the observed catalytic efficiency rather than impair enzyme function.

Finally, the detectable cellulolytic activity of intact recombinant cells demonstrated the feasibility of using engineered *S. cerevisiae* as a whole-cell biocatalyst. These results highlight the robustness of EglS and support its potential application in yeast-based platforms for cellulose hydrolysis and future consolidated bioprocessing.

## Figures and Tables

**Table 1:** Microbial strains and plasmids used in this study.

Strain	Relevant Features	Source or References
*S. cerevisiae* EBY100	*MAT***a***AGA1*::*GAL1-AGA1::URA3 ura3-52 trp1 leu2*D*1his3*D*200 pep4*::*HIS3 prb1*D*1.6R can1 GAL.*	ATCC (MYA4941) [17]
*S. cerevisiae* JARC06	EBY100 with pITD06.	This study
*E. coli* JARC03	*E. coli* DH5a [*endA1 hsdR17(*r_K_^−^m_K_^+^)*supE44 thi-1 recA1pyrA*(Nal^r^) *relA1*D(*lacIAA-argF*)*U169deoR* [f80d*lac*D(*lacZ*)*M15*] with pITD03; Amp^r^.	[16]
Plasmid		
pGEM-T Easy Vector	3.015 kb cloning vector, (pUC/pMB1 derived), MCS, lacZ α complementation, T7 and SP6 RNA-pol promoters; Amp^r^.	(Promega)
pYD1	5.0 kb expression vector, AGA2 gene, GAL1 promoter, Xpress and V5 epitopes, 6xHis tag, TRP1 gene, CEN6/ARS4, pUC ori; Amp^r^.	[18]
pITD03	pUCIDT carrying *B. subtilis egl*S ORF; Amp^r^.	[16]
pITD06	pYD1 carrying a 1.4 kb *Bam*HI-*Not*I PCR fragment from pITD03; Amp^r^.	This study

**Table 2:** Endoglucanase activity in different fractions of *Saccharomyces cerevisiae*.

Sample	Protein Concentration (mg mL^−1^)	Protein in Assay (mg)	Reducing Sugars (mg mL^−1^) *	Total Sugars Produced (mg)	Enzyme Activity (U) **	Volumetric Activity (U mL^−1^ Extract) ***	Total Activity per Fraction (U) ****	Specific Activity (U mg^−1^ Protein)
Cytoplasmic extract	1.97 ± 0.11	0.099 ± 0.0055	0.379 ± 0.0048	1.895 ± 0.024	0.526 ± 0.007	10.52 ± 0.13	52.6 ± 0.7	5.33 ± 0.31
Cell wall-associated proteins	0.065 ± 0.011	0.0033 ± 0.0006	0.137 ± 0.0016	0.683 ± 0.008	0.190 ± 0.002	3.79 ± 0.04	18.95 ± 0.2	58.4 ± 10.1
Whole recombinant cells	—	—	0.057 ± 0.0000	0.287 ± 0.000	0.080 ± 0.000	1.59 ± 0.00 †	—	—

* Reducing sugars were quantified as glucose equivalents using the DNS assay. ** One unit (U) of enzyme activity was defined as the amount of enzyme releasing 1 µmol of glucose equivalents per minute under the assay conditions (based on reducing sugars released during a 500 µL reaction containing 50 µL of enzyme sample, prior to DNS addition). *** Volumetric activity (U mL^−1^ extract) was calculated based on the activity measured using 50 µL of sample. **** Total activity was calculated considering a final extract volume of 5 mL. † For whole recombinant cells, volumetric activity was calculated based on the equivalent assay volume (50 µL of cell suspension). Cells from a 50 mL culture were resuspended in 5 mL of PBS prior to DTT treatment, and aliquots (50 µL) were used for activity assays. Protein concentration and specific activity were not determined.

## Data Availability

The original contributions presented in this study are included in the article. Further inquiries can be directed to the corresponding authors.
